# Bilateral Renal Tumors in Children: The First 5 Years’ Experience of National Centralization in The Netherlands and a Narrative Review of the Literature

**DOI:** 10.3390/jcm10235558

**Published:** 2021-11-26

**Authors:** Sophie E. van Peer, Janna A. Hol, Alida F. W. van der Steeg, Martine van Grotel, Godelieve A. M. Tytgat, Annelies M. C. Mavinkurve-Groothuis, Geert O. R. Janssens, Annemieke S. Littooij, Ronald R. de Krijger, Marjolijn C. J. Jongmans, Marc R. Lilien, Jarno Drost, Roland P. Kuiper, Harm van Tinteren, Marc H. W. A. Wijnen, Marry M. van den Heuvel-Eibrink

**Affiliations:** 1 Department of Pediatric Oncology, Princess Máxima Center for Pediatric Oncology, Heidelberglaan 25, 3584 CS Utrecht, The Netherlands; j.hol@prinsesmaximacentrum.nl (J.A.H.); a.f.w.vandersteeg@prinsesmaximacentrum.nl (A.F.W.v.d.S.); m.vangrotel@prinsesmaximacentrum.nl (M.v.G.); g.a.m.tytgat@prinsesmaximacentrum.nl (G.A.M.T.); A.M.C.mavinkurve-groothuis@prinsesmaximacentrum.nl (A.M.C.M.-G.); g.o.r.janssens@prinsesmaximacentrum.nl (G.O.R.J.); a.s.littooij-2@prinsesmaximacentrum.nl (A.S.L.); r.r.dekrijger-2@prinsesmaximacentrum.nl (R.R.d.K.); m.c.j.jongmans-3@umcutrecht.nl (M.C.J.J.); m.lilien@umcutrecht.nl (M.R.L.); j.drost@prinsesmaximacentrum.nl (J.D.); r.kuiper@prinsesmaximacentrum.nl (R.P.K.); h.vantinteren@prinsesmaximacentrum.nl (H.v.T.); m.h.w.wijnen-5@prinsesmaximacentrum.nl (M.H.W.A.W.); m.m.vandenheuvel-eibrink@prinsesmaximacentrum.nl (M.M.v.d.H.-E.); 2 Department of Radiation Oncology, University Medical Center Utrecht (UMCU), Heidelberglaan 100, 3584 CX Utrecht, The Netherlands; 3 Department of Radiology and Nuclear Medicine, Wilhelmina’s Children Hospital, University Medical Center Utrecht (UMCU), Heidelberglaan 100, 3584 CX Utrecht, The Netherlands; 4 Department of Pathology, University Medical Center Utrecht (UMCU), Heidelberglaan 100, 3584 CX Utrecht, The Netherlands; 5 Department of Genetics, University Medical Center Utrecht (UMCU), Heidelberglaan 100, 3584 CX Utrecht, The Netherlands; 6 Department of Pediatric Nephrology, Wilhelmina’s Children Hospital, University Medical Center Utrecht (UMCU), Heidelberglaan 100, 3584 CX Utrecht, The Netherlands; 7 Oncode Institute, Heidelberglaan 25, 3584 CS Utrecht, The Netherlands

**Keywords:** bilateral, stage V, Wilms tumor, RCC, pediatric, renal tumor

## Abstract

Survival of unilateral Wilms tumors (WTs) is exceeding 90%, whereas bilateral WTs have an inferior outcome. We evaluated all Dutch patients with bilateral kidney tumors, treated in the first five years of national centralization and reviewed relevant literature. We identified 24 patients in our center (2015–2020), 23 patients had WT/nephroblastomatosis and one renal cell carcinoma. Patients were treated according to SIOP-RTSG protocols. Chemotherapy response was observed in 26/34 WTs. Nephroblastomatosis lesions were stable (*n* = 7) or showed response (*n* = 18). Nephron-sparing surgery was performed in 11/22 patients undergoing surgery (*n* = 2 kidneys positive margins). Local stage in 20 patients with ≥1 WT revealed stage I (*n* = 7), II (*n* = 4) and III (*n* = 9). Histology was intermediate risk in 15 patients and high risk in 5. Three patients developed a WT in a treated nephroblastomatosis lesion. Two of 24 patients died following toxicity and renal failure, i.e., respectively dialysis-related invasive fungal infection and septic shock. Genetic predisposition was confirmed in 18/24 patients. Our literature review revealed that knowledge is scarce on bilateral renal tumor patients with metastases and that radiotherapy seems important for local stage III patients. Bilateral renal tumors are a therapeutic challenge. We describe management and outcome in a national expert center and summarized available literature, serving as baseline for further improvement of care.

## 1. Introduction

Renal tumors account for approximately 6% of all childhood cancers [1,2]. Unilateral Wilms tumors comprise the largest subgroup of childhood renal tumors (85–90% of pediatric renal tumor patients) and survival rates of these patients are currently exceeding 90% [3,4].

Bilateral or stage V renal tumors represent a rare condition and mainly include Wilms tumors. They comprise approximately 5–8% of all Wilms tumors and may present synchronously, or metachronously [1,3,5,6]. Whereas survival rates of patients with unilateral Wilms tumor are high, survival of patients with bilateral Wilms tumors seems highly variable, ranging from 43–91% [3,7,8]. Long-term outcomes are expected to be even less favorable, based on tumor behavior, but also due to disease- and treatment-related kidney disease with its subsequent comorbidity and corresponding lower life expectancy [9,10,11,12,13]. In addition, patients with bilateral renal tumors more frequently carry underlying genetic predisposition than patients with unilateral tumors [5]. These include patients with germline WT1-mutations or deletions, which induce an increased risk of (end-stage) syndrome-related kidney disease [14].

Management of patients with bilateral renal tumors often includes dilemmas in therapeutic decision making, reflecting the balance between aiming for complete remission of cancer and preservation of kidney function. Recent International Society of Pediatric Oncology Renal Tumor Study Group (SIOP-RTSG) protocols (SIOP 93-01, SIOP 2001 and the SIOP-RTSG 2016 UMBRELLA protocol) and the Children’s Oncology Group (COG) protocols, recommend preoperative chemotherapy for bilateral Wilms tumor patients to decrease tumor size. When feasible and oncologically safe, this is followed by nephron-sparing surgery (NSS), preferably for both kidneys, to preserve as much remnant healthy renal tissue as possible [3,14]. However, although overall survival rates have increased over the past decades, patients with bilateral Wilms tumors still suffer from relatively high relapse rates as well as a significant risk of kidney disease at follow-up. To preserve as much kidney tissue as possible, individual treatment decisions have to be made in many cases. For this purpose, international collaboration within multidisciplinary expert panels in this field is highly aimed for in order to benefit from international expertise and experience, as well as to learn from these complicated patients for the future. Therefore, referral to, or consultation of, surgical expert centers as defined in ExPO-r-Net (http://www.expornet.eu/, accessed on 23 November 2021) is highly recommended for patients with bilateral renal tumors in SIOP-RTSG settings [3].

In The Netherlands, centralization of pediatric cancer care was initiated in November 2014 in the Princess Máxima Center for Pediatric Oncology, with, from the start, the aim of centralized multidisciplinary management of all pediatric oncology cases, including all renal tumor cases. Here, we describe the characteristics, management and outcome of the registered pediatric bilateral renal tumor patients in the first five years of centralization. This serves as a baseline for further improvement, with the background perspective of a review of all available bilateral renal tumor series in literature.

## 2. Materials and Methods

### 2.1. Patients

We retrospectively reviewed all patients with bilateral de novo renal tumors at presentation, from the 1st January 2015 until the 31st of December 2019, that were diagnosed in the Princess Máxima Center for Pediatric Oncology. We included patients younger than 19 years that presented with any synchronous or metachronous bilateral renal masses/suspicious abnormalities on radiographic imaging. Patient demographics, diagnostic information, clinical characteristics, administered treatment(-modalities), tumor characteristics and clinical follow-up were recorded if informed consent was provided (EudraCT numbers 2007-004591-39, 2016-004180-39, MEC 202.t34/2001/122, MEC-2018-026).

Hypertension at diagnosis was assessed according to the 2016 European Society of Hypertension guideline for children and adolescents [15]. All patients were treated according to the SIOP 2001 or the SIOP-RTSG 2016 UMBRELLA protocol [3,16]. Diagnostic imaging and response assessment consisted of magnetic resonance imaging including diffusion-weighted imaging (MRI-DWI), abdominal ultrasound and chest computed tomography scan (CT-scan). All patients were treated after central radiology review by a member of the SIOP-RTSG radiology panel. Treatment decisions for individual bilateral renal tumor patients were further made by consensus in the multidisciplinary tumor board.

Preoperative chemotherapy regimens were, if necessary, adapted according to volume response, based on radiology. We retrospectively assessed response to chemotherapy based on tumor volume measured on MRI-DWI, using the following formula to calculate the tumor volume: 0.523 times the product of the three tumor dimensions [3]. Response was defined as a decrease of tumor volume of more than 10%, as previously described [17]. 

It was aimed to perform NSS on both kidneys or, if not feasible, complete nephrectomy combined with contralateral enucleation or partial nephrectomy. Surgical recommendations according to SIOP-RTSG [3] and central review of histology slides by a reference SIOP-RTSG panel pathologist were considered standard of care [18,19]. Depending on stage and histological type/risk group assignment, patients were postoperatively treated with chemotherapy and if necessary, radiotherapy. The latter cases were all treated with novel radiotherapy modalities such as intensity-modulated radiotherapy (IMRT) as recently reported [20]. 

Patients were closely monitored after end of treatment according to SIOP-RTSG follow-up recommendations. Kidney function was monitored and estimated glomerular filtration rate (eGFR) at follow-up was retrospectively calculated based on creatinine levels using the modified Schwartz-formula for children [21]. All bilateral renal tumor patients were offered referral to a clinical geneticist as standard of care.

### 2.2. Literature Review

In order to compare our results with existing available historical bilateral renal tumor series, we searched PubMed and Embase databases using different synonyms for the words ‘Wilms tumor’ and ‘bilateral’ and performed reference checking of the included articles to identify all reported series until 1st of June 2021. The search terms are specified in Supplementary Table S1. We included all case series in English describing more than five pediatric patients with synchronous or metachronous bilateral Wilms tumors and extracted all clinical, therapeutic and outcome data, and we specifically searched for bilateral renal tumor patients with metastases. We checked all papers for multiple reports describing possible overlapping cohorts and only included the paper with the largest number of patients, most recent publication or most comprehensive description of patients.

## 3. Results

### 3.1. Subsection

Over the period of five years, we identified 125 patients with Wilms tumor/nephroblastomatosis lesions, of which 25 patients presented with bilateral disease (19.2%) in the Princess Máxima Center for pediatric oncology (Table 1 and Table S2). One of these patients’ parents refused to provide informed consent for registration of anonymized clinical data. Of the remaining 24 patients, 20 had at least one Wilms tumor (unilateral Wilms tumor with contralateral nephroblastomatosis or bilateral Wilms tumors with or without nephroblastomatosis), of which six kidneys of six patients contained multiple Wilms tumors. Three patients had nephroblastomatosis/nephrogenic rests only (without Wilms tumor). In total, 34 Wilms tumors were detected in these 24 patients. One previously reported 15-year-old girl had a renal cell carcinoma (RCC) with a contralateral localized conglomerate of renal cystic masses [22].

**Figure 1:**
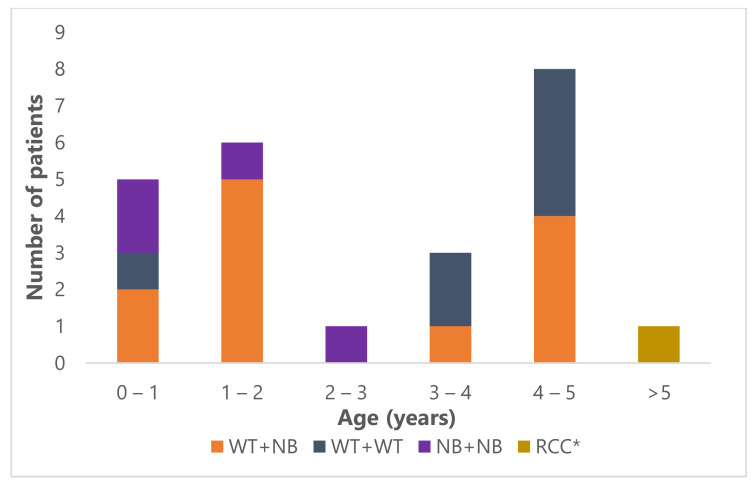
Age distribution of the 24 bilateral renal tumor patients. WT: Wilms tumor; NB: nephroblastomatosis; RCC: renal cell carcinoma; *: the patient with RCC was 15 years old at diagnosis.

Median age at presentation of the 20 Wilms tumor patients was 38 months (range 7–59 months) and of the patients with nephroblastomatosis only, 24 months (range 12–25 months) (Figure 1). Eleven of 23 patients were male (45.8%).

Presenting symptoms are described in Supplementary Table S3. Hypertension at presentation was observed in 16/23 Wilms tumor/nephroblastomatosis patients (69.6%) (14 patients with at least one Wilms tumor, two patients with bilateral nephroblastomatosis only). One patient had hypertension with severe headaches at diagnosis, requiring admission to the pediatric intensive care unit (PICU) for intravenous antihypertensive treatment and monitoring. The RCC patient was normotensive at diagnosis.

### 3.2. Diagnostic Imaging

All patients underwent an abdominal ultrasound, abdominal MRI-DWI and chest CT-scan at diagnosis for initial staging of the tumor (localized or metastasized/bilateral disease). Three of all 24 patients presented with metastases at diagnosis and one of 24 developed metastasis during treatment (patient 8 in Supplementary Table S2). Median Wilms tumor volume at diagnosis on MRI-DWI was 103 mL (interquartile range (IQR) 9.68–573.25 mL). Median Wilms tumor volume after preoperative chemotherapy on MRI (or ultrasound when MRI was unavailable) was 23.8 mL (IQR 1.95–203.8 mL). The RCC volume at diagnosis was 2246 mL. Two patients (aged 12 and 51 months) were biopsied before starting preoperative chemotherapy (1 case to confirm suspicion of nephroblastomatosis only, the other (Wilms tumor) had an aspecific presentation with osseous metastases near the neuroforamen).

### 3.3. Preoperative Chemotherapy and Response

**Figure 2:**
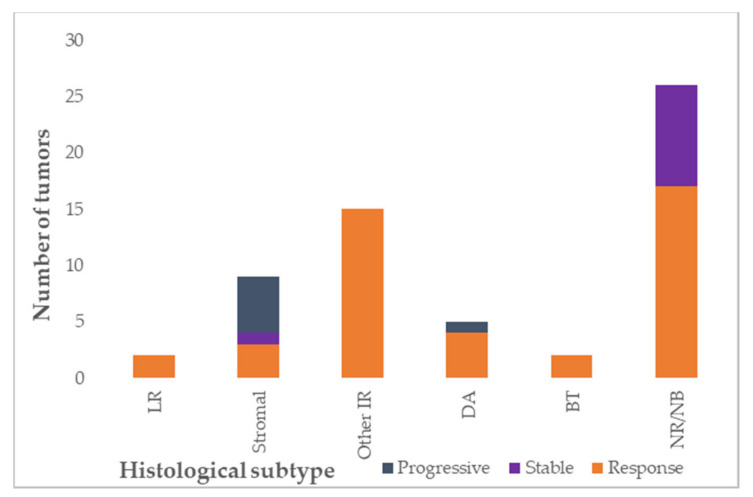
Response to preoperative chemotherapy according to histological subtype, depicted per lesion (total evaluable *n* = 58, Wilms tumor = 33, nephrogenic rests *n* = 25). LR: low-risk; IR: intermediate-risk; DA: diffuse anaplasia; high-risk blastemal type; NR: nephrogenic rest; NB: nephroblastomatosis. Response: >10% volume reduction of tumor, stable: less than 10% increase or decrease in tumor volume, progressive: >10% increase in tumor volume.

All 23 Wilms tumor/nephroblastomatosis patients received preoperative chemotherapy according to the SIOP 2001/SIOP-RTSG 2016 UMBRELLA protocols. This consisted of vincristine 1.5 mg/m^2^ and actinomycin-D 45 µg/kg, with or without doxorubicin 50 mg/m^2^. Doxorubicin was added for patients with metastasized disease at diagnosis, or in patients with unresponsive disease after 4–6 weeks of preoperative treatment in the SIOP 2001 protocol. In case of unresponsive disease after 4–6 weeks of preoperative treatment in the SIOP-RTSG 2016 UMBRELLA protocol, vincristine/actinomycin-D treatment was replaced by carboplatin/etoposide [3,16]. Chemotherapy was administered for a median duration of six weeks (range 4–12 weeks) before the first surgery. Detailed information on response to preoperative chemotherapy on MRI could be assessed in all patients that received (preoperative) chemotherapy treatment. In total, 34 Wilms tumors were detected in 20 patients, six patients had multiple tumors in one kidney. A response to preoperative chemotherapy was observed in 26 tumors in 18 patients (low-risk tumors *n* = 2, stromal tumors *n* = 3, other intermediate-risk *n* = 15, diffuse anaplastic tumors *n* = 4, blastemal tumors *n* = 2). A decrease less than 10% was found in one stromal Wilms tumor, and progression in six tumors in five patients (five stromal Wilms tumors, and one diffuse anaplastic Wilms tumor) (Table 1 and Table S2, Figure 2). For one (regressive type) Wilms tumor, post-hoc analysis of radiologic response to chemotherapy was not possible since the tumor was too small for reliable measurements. Five patients revealed response in one tumor, but progression, stable disease or no response measurement in another tumor (Supplementary Table S2). Details on response according to histology are depicted in Figure 2.

Thirty-nine lesions in 21 patients were suspected of nephroblastomatosis on MRI-DWI (27 histologically confirmed, type was perilobar *n* = 21, intralobar *n* = 5, both intra- and perilobar nephrogenic rests *n* = 4, not otherwise specified and tumor-like nephrogenic rest *n* = 9). Response information was available for 25/39 nephroblastomatosis lesions. Seven of these 25 lesions did not change upon treatment with chemotherapy (one intralobar, three combined nephrogenic rests and three not otherwise specified lesions) and 18 showed a decrease in size after preoperative chemotherapy (10 perilobar, two intralobar, and six not otherwise specified lesions). 

Three patients without metastases received additional doxorubicin during chemotherapy before any surgery, because of unsatisfactory response on imaging (according to the SIOP 2001 protocol). This led to a decrease in tumor volume in only one of them (regressive type Wilms tumor). Two other patients (both stromal-type Wilms tumors) received additional carboplatin/etoposide (according to SIOP-RTSG 2016 UMBRELLA), due to unresponsive disease on imaging, after which both patients did not show a significant decrease of tumor size. 

All four metastatic stage V patients received upfront vincristine/actinomycin-D/doxorubicin. Two of these patients with metastases revealed a decrease of >10% in both kidneys (patients 6 and 7 in Supplementary Table S2), one patient showed response in one kidney (regressive type Wilms tumor) and stable disease in the other kidney (nephroblastomatosis, patient 19 in Supplementary Table S2) and one patient experienced a response in one kidney, but progression in the other kidney (both diffuse anaplastic Wilms tumors, patient 8 in Supplementary Table S2).

### 3.4. Surgery

**Figure 3:**
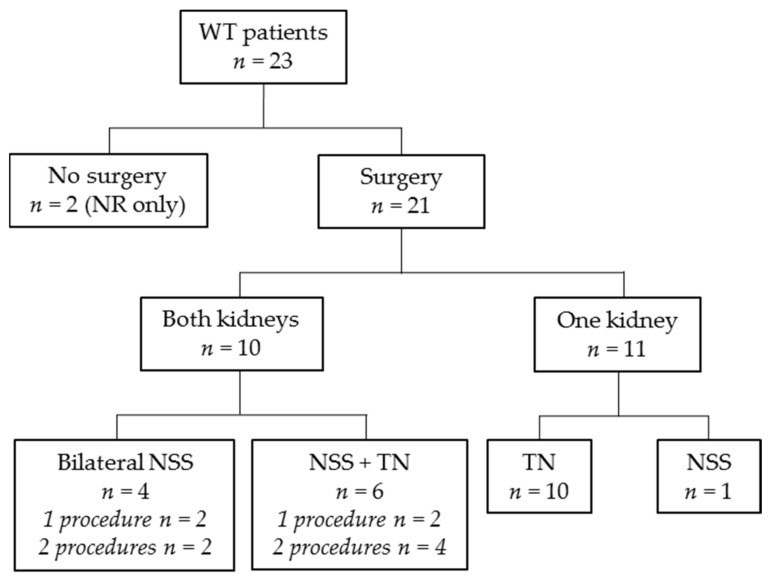
Flow diagram revealing applied surgery modality. WT: Wilms tumor; n: number of patients; NR: nephrogenic rests; NSS: nephron-sparing surgery; TN: tumor nephrectomy.

Abdominal surgery was performed in 21/23 Wilms tumor/nephroblastomatosis patients (Figure 3). Two patients did not undergo surgery after MRI-DWI as their lesions were highly suspect for bilateral nephroblastomatosis (confirmed with biopsy in one of two patients). Sixteen patients underwent one surgery procedure, five underwent two procedures. Ten of 21 patients who underwent surgery, underwent unilateral nephrectomy only (three with contralateral biopsy), and 11/21 patients underwent NSS. Details on surgical procedures per kidney are depicted in Table 1 and Figure 3. Of the five synchronous Wilms tumor patients that underwent separate surgeries in multiple procedures on both kidneys, the largest tumor was removed first. These five patients received additional chemotherapy in between surgical procedures (vincristine/actinomycin-D/doxorubicin (*n* = 3), vincristine (*n* = 1) and cyclophosphamide/doxorubicin (*n* = 1)). This led to a preoperative chemotherapy duration for the second kidney of more than 12 weeks in two patients (both had preoperative chemotherapy for a total of 13 weeks). The other three patients with additional chemotherapy in between surgeries had a total of 10 (*n* = 2) or 11 (*n* = 1) weeks of preoperative chemotherapy. This did not lead to additional response in the remaining tumor in any of the cases, nor to progression of disease or relapse.

### 3.5. Local Stage and Histology

**Figure 4:**
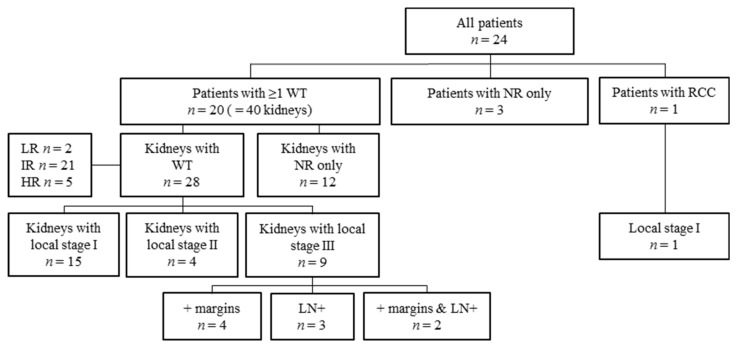
Flow diagram of stage and histology per kidney. n: number; WT: Wilms tumor, NR: nephrogenic rests; RCC: renal cell carcinoma; LR: low-risk histology; IR: intermediate-risk histology; HR: high-risk histology; + margins: positive resection margins; LN+: lymph nodes positive for tumor cells.

Histology and staging per patient and kidney are depicted in Figure 4. According to the highest risk histology type, 15/20 patients with at least one Wilms tumor had intermediate-risk histology, five patients had high-risk histology and no patients revealed low-risk histology (Table 1 and Table S2). Of 16 non-metastatic patients with at least one Wilms tumor, highest local abdominal stage was stage I disease (*n* = 7), stage II (*n* = 4) and stage III (*n* = 5), according to the SIOP staging criteria [18,19]. Stage III was based on positive resection margins (*n* = 4) or lymph nodes with vital tumor cells or chemotherapy effect (*n* = 1).

The four metastatic patients all had local stage III disease (patient 6, 7, 8 and 19 in Supplementary Table S2), based on lymph nodes with therapy effect (*n* = 2), or both positive lymph nodes and microscopically positive resection margins (*n* = 2). Positive microscopic margins were observed in two (13.3%) of the 15 kidneys following NSS and in 4/16 (25%) kidneys that were completely removed.

In four patients, histological discrimination between nephrogenic rest and stage I Wilms tumor was not possible, even after a second independent international review assessment by the international SIOP-RTSG pathology panelist. These four patients all had Wilms tumor in the contralateral kidney and were treated based on the stage and histology of the Wilms tumor and received chemotherapy (vincristine/actinomycin-D) up to one year for nephroblastomatosis treatment.

### 3.6. Postoperative Treatment

All 21 Wilms tumor patients who underwent surgery, received postoperative chemotherapy according to the aforementioned SIOP-RTSG protocols [3,16]. All 21 patients with nephroblastomatosis in one or both kidneys received additional monthly chemotherapy (vincristine/actinomycin-D) up to a total of one year.

All local stage III disease patients received post-operative flank irradiation, using IMRT [20,23]. Metastatic sites were irradiated in two patients with bone (vertebral) lesions and in two with pulmonary metastases. The lung lesions of one of the patients with bone lesions had completely resolved by preoperative chemotherapy at time of surgery and were therefore not irradiated (patient 19, regressive type Wilms tumor).

### 3.7. Toxicity and Outcome

Four patients needed unplanned pediatric intensive care unit (PICU) admission. One patient was admitted to the PICU in the preoperative phase due to respiratory insufficiency based on large abdominal tumor mass (unresponsive to chemotherapy) (patient 14, Supplementary Table S2). This patient underwent surgery after two preoperative chemotherapy courses (stromal type). He is currently well and in complete remission, without any sign of end-stage kidney disease (ESKD) (patient 14 in Supplementary Table S2). Extensive treatment-related toxicity led to PICU admission in the other three patients. One patient experienced postoperative intra-abdominal fluid leakage, leading to metabolic acidosis and PICU admission. Second look surgery revealed no cause for fluid leakage and the patient recovered after four days. Another patient was admitted to the PICU nine weeks into high-risk therapy for blastemal Wilms tumor due to acute kidney injury (AKI) stage III, during a period of bacterial sepsis. He was later (after finalizing treatment) readmitted to the PICU for hematemesis and seizures secondary to hypertension. The latter was caused by extensive large intestine necrosis while suffering from systemic candida infection. This patient deceased five months after finalizing high-risk chemotherapy treatment, after having been on peritoneal dialysis (that started three months after surgery) for a year. The third patient experienced respiratory insufficiency during preoperative treatment (most likely due to fluid overload and large tumor mass) and was later re-admitted to the PICU for AKI that presented seven months post-surgery during high-risk treatment for diffuse anaplastic Wilms tumor. This patient deceased in the period between two surgeries, due to circulatory shock while on dialysis.

Median follow-up for the 23 stage V Wilms tumor/nephroblastomatosis patients is 34 months (range 12–74 months) from diagnosis, at the moment of data collection. In this follow-up period, three patients developed a localized Wilms tumor in a previously treated nephroblastomatosis lesion (perilobar nephrogenic rest (*n* = 1), intralobar nephrogenic rest (*n* = 1) and nephrogenic rest not otherwise specified (*n* = 1)), 5, 13 and 17 months after finalizing chemotherapy for these lesions, respectively. All three had a confirmed predisposition, i.e., a germline WT1-mutation (*n* = 2) and hypermethylation of the H19-locus in healthy kidney and tumor tissue only (*n* = 1). These patients subsequently received treatment for the new Wilms tumor. To date, only one has undergone surgery for the second Wilms tumor, revealing a stromal Wilms tumor.

Two patients in our cohort died, as described above, mainly based on treatment-related toxicity. Both died after development of ESKD and being on dialysis. One other patient has chronic kidney disease (CKD) stage 2 at moment of data collection based on eGFR <90 mL/min/1.73 m^2^ (follow-up time 27 months). All 22 other patients have excellent eGFR (>90 mL/min.1.73 m^2^ at last follow-up). Only one patient developed hypertension during follow-up, however at that moment she was treated for a second Wilms tumor, appearing from a nephrogenic rest. Proteinuria was not systematically measured during follow-up in our cohort. All patients are under strict nephrological follow-up according to the surveillance guideline of the SIOP-RTSG 2016 UMBRELLA protocol, including regular blood pressure monitoring, extensive urine and blood analyses and eGFR measurements.

### 3.8. Predisposition

All bilateral Wilms/nephroblastomatosis patients’ parents accepted the offered referral to a clinical geneticist as standard of care, or were already under surveillance, because of a predisposition syndrome. In 17/23 a genetic predisposition was confirmed, in three patients already before presentation of Wilms tumor/nephroblastomatosis (Beckwith-Wiedemann syndrome (*n* = 1), WAGR syndrome (*n* = 1) and hemihypertrophy not otherwise specified (*n* = 1)). In the current series, apart from standard of care genetic counseling, all applicable patients were invited to participate in a comprehensive genetic predisposition prospective genotype-phenotyping study including whole-exome sequencing and whole-genome sequencing. A comprehensive and detailed description of the methods and results of this genetic predisposition study of all (including unilateral) Wilms tumor patients diagnosed in this period will be reported separately.

### 3.9. Literature Review of All Reported Cases including the Current Cohort 

After a literature search in PubMed and Embase and cross-reference checking, we identified 36 papers reporting >5 bilateral Wilms tumor/nephroblastomatosis patients. This included a total of 1497 patients, including our series (Table 2, Supplementary Figure S1). Four of these papers were large series with more than 100 stage V patients [7,24,25,26]. Median age of these cohorts was 12–39 months, and sex was reported in 1136 patients (41% male). Seven papers reported information on blood pressure at diagnosis, identifying hypertension in 80/240 patients (33.3%).

Preoperative chemotherapy was administered in 88.7% of patients with bilateral disease. Information on response to chemotherapy was reported in 783 patients, of which 163 showed no response (histological subtype not specified *n* = 139 (85.3%), stromal type *n* = 15 (9.2%), diffuse anaplasia *n* = 5 (3.1%) and cystic partially differentiated nephroblastoma (CPDN)/epithelial type *n* = 4 (2.5%)).

Information on surgical modality was available for 1048 patients. Of these, 358 (34%) underwent bilateral NSS, 158 (15.1%) unilateral nephrectomy, 408 (38.9%) NSS combined with contralateral total nephrectomy, 46 (4.4%) unilateral NSS and 28 (2.7%) bilateral total nephrectomy. Forty-eight patients did not undergo surgery due to death during preoperative chemotherapy (*n* = 7), due to nephroblastomatosis only (*n* = 3), because of inoperable tumors (*n* = 1) or reason for withholding of surgical treatment not specified (*n* = 37). NSS was performed in at least one kidney in 812/1048 patients with reported information on type of surgery. Of these, information on resection margins was only available for 142 kidneys, of which 22 reported (microscopic) positive margins after NSS (15.5%). Radiotherapy administration was reported for 359 patients (preoperative in nine patients, postoperative in 319 patients and moment unknown in 31 patients). Two reports described a total of 40 patients with local stage III who did not receive postoperative radiotherapy [24,27,28]. Both papers suggest the occurrence of a higher number of relapses for stage III patients that were not irradiated, or a lower overall survival [24,27]. Kieran et al. compared renal outcome between the irradiated and non-irradiated patients in their series, and they did not observe a significant loss of renal function in the irradiated group versus the non-irradiated group [29].

Metastases were reported in 188 of 1409 patients (13.3%) with information on localized or metastasized disease. Histology of stage V patients with metastases was only reported in 16 patients and was diffuse anaplasia in three patients, anaplasia not otherwise specified in three patients, unfavorable histology not otherwise specified in three patients, favorable histology not otherwise specified in three patients, blastemal in three patients and regressive in one patient. Twenty-four of 35 patients with bilateral and metastatic disease with information on outcome, died (68.6%). Details on these patients with stage V and metastatic disease are presented in Supplementary Table S4.

Of 866 patients for which information on functional renal outcome was reported, 76 developed ESKD during follow-up (8.8%, varying in the reports from 3–54%). Outcome information regarding relapse was available of 876 patients (29 papers), of which 160 (18.2%) developed a relapse during follow-up. Outcome information regarding survival was available for 1297 patients, of which 255 died (19.7%). Survival of patients reported in more recent studies tended to be more favorable compared with older studies, however exact comparison is difficult due to small series and differences in follow-up time.

**Table 2:** Literature review.

Author, Year	No. of pt	Med Age (mths)	Sex		HTN at Dx (%)	Meta (%)	Preop CT		No CT Response (Hist Type)	Surgery Type					+ Margins after NSS (% Kidneys)	RTx (Postop)	Postop CT	ESKD (%)	NB		Outcome		OS %
			M	F			Yes	No		bNSS	uTN	uTN + uNSS	uNSS	bTN					Yes (%)	No	Relapse	Death	
Aydin, 2019 [30]	30	21.7	13	17	NA	3 (10)	30	0	7 (4 CPDN/ET, 3 DA)	6	3	19	1	1	3/32 (9)	4 * 6	NA	1 (3.3)	6 (20)	24	NA	NA	86 (10y)
Doganis, 2019 [31]	21	3.1 y	11	10	NA	0	21	0	NA	NA	NA	NA	NA	NA	NA	NA	NA	NA	4 (19)	17	3	4	76.3 (5y)
Saha, 2019 [32]	6	2 y	2	4	NA	0	6	0	0	6	0	0	0	0	NA	NA	NA	0	NA	NA	2	0	90 (2–5y)
Tan, 2018 [33]	18	2.28 y	10	8	NA	NA	18	0	NA	16	0	2	0	0	1/34 (3)	NA	NA	0	NA	NA	2	2	85.56 (4y)
Ehrlich, 2017 [7]	189	NA	NA	NA	NA	27 (14)	189	0	60 (NA)	66	20	90	8	5	NA	NA	NA	NA	NA	NA	23	1	94.9 (4y)
Chen, 2016 [34]	7	12	3	4	NA	NA	5	2	NA	0	5	2	0	0	NA	3	7	2 (29)	2 (29)	5	2	0	NA
Davidoff, 2015 [35]	42	2.2 y	19	23	NA	7 (17)	42	0	NA	39	0	3	0	0	NA	18	40	0	NA	NA	7	6	85.7 (3y)
Agarwala, 2014 [36]	11	6–30	8	3	NA	3 (27)	11	0	NA	7	0	3	0	0	NA	6	10	1 (9.1)	NA	NA	5	1	90 (5y)
Hubertus, 2015 [37]	22	39.4	10	12	NA	4 (18)	21	1	NA	10	0	12	0	0	NA	3	NA	NA	17 (85)	5	3	2	NA
Furtwängler, 2014 [24]	136	2.5 y	NA	NA	NA	19 (14)	104	17	13 (NA)	43	15	33	11	0	30%	16	116	NA	NA	NA	27	20	NA
Oue, 2014 [38]	31	15.5	16	15	NA	2 (6)	24	7	3 (NA)	10	0	15	0	3	NA	3 *1	NA	4 (16)	NA	NA	3	2	92.6 (5y)
Hadley, 2013 [39]	20	2.5 y	11	9	NA	4 (20)	20	0	NA	NA	NA	NA	NA	NA	3/22 (14)	13	NA	2 (10)	NA	NA	2	3	85 (2y)
Indolfi, 2013 [27]	93	24	32	61	NA	11 (12)	81	6	NA	35	17	31	5	1	NA	20	NA	NA	6 (6)	85	27	17	80
Sulkowski, 2012 [40]	12	17	4	8	NA	1 (8)	10	2	NA	6	0	5	1	0	NA	3	NA	0	NA	NA	0	0	NA
Sudour, 2012 [28]	49	2.3 y	18	31	10 (20)	5 (10)	49	0	11 (10 ST, 1 DA)	19	0	29	0	1	NA	11	44	7 (14.3)	NA	NA	7	5	89.5 (5y)
Halim, 2012 [41]	25	34.5 (m)	9	16	6 (24)	2 (8)	25	0	6 (not ST)	2	3	13	0	0	NA	3	NA	0	NA	NA	5	9	74 (3y)
Millar, 2011 [42]	23	2.29 y	8	15	12 (52)	1 (4)	22	1	NA	NA	NA	NA	NA	NA	NA	2	NA	NA	19 (83)	4	7	11	55.6 (5y)
Hamilton, 2011 [25]	188	32	74	114	NA	16 (18)	105	83	1 (NA)	64	6	104	8	6	NA	64	NA	23 (12)	58 (31)	130	54	NA	84 (8y)
Weirich, 2004 [43]	28	1.9 y	NA	NA	NA	3 (11)	28	0	NA	NA	NA	NA	NA	NA	NA	NA	NA	15 (54)	NA	NA	5	4	NA
Kubiak, 2004 [44]	23	19	7	16	9 (39)	4 (17)	18	5	15 (NA)	5	0	18	0	0	8/28 (29)	NA	NA	2 (9)	8(35)	15	NA	6	NA
Cooper, 2000 [45]	23	30.6 (m)	8	15	NA	NA	21	2	NA	NA	NA	NA	NA	NA	NA	NA	NA	NA	NA	NA	9	10	NA
Fuchs, 1999 [46]	14	22	2	12	NA	0	11	1	6 (NA)	9	1	4	0	0	NA	NA	NA	0	NA	NA	1	1	NA
Kullendorff, 1999 [47]	6	2.4 y (m)	1	5	NA	NA	6	1	1 (NA)	3	2	0	0	0	NA	2	1	NA	2 (33)	5	1	2	NA
Nawaz, 1999 [48]	7	3 y (m)	5	2	NA	NA	5	2	0	3	1	1	0	0	NA	2	5	NA	NA	NA	1	3	NA
Tomlinson 1999 [49]	8	1.1 y	3	5	NA	1	7	1	NA	1	3	2	2	0	0/8	1	8	0	6 (75)	2	0	2	NA
Kumar, 1998 [50]	70	24.4 (m)	28	42	32 (45)	9 (13)	57	13	34 (NA)	11	10	30	4	6	NA	21	NA	3 (4)	22 (31)	48	NA	22	NA
Alfer, 1993 [51]	14	37	2	12	NA	0	14	0	NA	8	0	5	0	0	5/11 (45)	2 *2	NA	NA	6 (43)	2	4	3	NA
Shearer, 1993 [52]	36	27	13	16	NA	4 (11)	NA	NA	NA	NA	NA	NA	NA	NA	NA	NA	NA	1 (3)	22 (61)	8	NA	4	64 (5y)
Montgomery, 1991 [26]	185	2.1 y (m)	80	105	NA	35 (19)	185	0	NA	NA	NA	NA	NA	NA	NA	101	NA	10 (5)	NA	NA	NA	51	73 (5y)
Coppes, 1989 [53]	67	20.2	26	41	NA	5 (7)	NA	NA	NA	NA	NA	NA	NA	NA	NA	NA	NA	NA	NA	NA	NA	24	64 (?y)
Hanash 1987 [54]	6	NA	3	3	NA	NA	NA	NA	NA	1	3	2	0	0	NA	1	6	NA	2 (33)	4	0	2	NA
Cohen, 1986 [55]	22	30	13	9	NA	4 (18)	NA	NA	NA	5	7	8	0	0	NA	18	20	2 (9)	9 (56)	7	NA	7	NA
Asch, 1985 [56]	21	28.8 (m)	6	15	4 (19)	3 (14)	NA	NA	NA	6	1	10	0	4	NA	11	NA	NA	NA	NA	3	9	57 (?y)
Wikström, 1982 [57]	6	12	6	0	1 (17)	NA	NA	NA	NA	2	2	2	0	0	NA	5	NA	NA	NA	NA	0	4	NA
Wasiljew, 1982 [58]	14	14.5	4	10	NA	NA	NA	NA	NA	3	4	7	0	0	NA	10	11	NA	NA	NA	2	5	NA
Jones, 1982 [59]	18	16	NA	NA	NA	4 (22)	NA	NA	NA	NA	NA	NA	NA	NA	NA	NA	NA	1	13 (72)	5	9	11	NA
Current cohort, 2021	23	31.5	11	12	16 (69.6)	4 (17)	23	0	6 (5 ST-WT, 1 DA)	6	4	9	6	1	2/15	7	23	2 (8.7)	21 (97.3)	2	0	2	NA
TOTAL	1491	12–39.4	466	670	80/240 (33.3%)	181/1409 (12.8%)	88.7%	11%	163/1307 (20.9%)	358	158	408	46	28	3–45%	-	359/1054 (34.1%)	3–54%	6–97.3%		18.2%	19.7%	

No.: number; pt: patients; Med: median; mths: months; M: male; F: female; HTN: hypertension; Dx: diagnosis; preop: preoperative; CT: chemotherapy; hist: histological; bNSS: bilateral nephron-sparing surgery; uTN: unilateral tumornephrectomy; uNSS: unilateral nephron-sparing surgery; bTN: bilateral tumornephrectomy; +: positive; RTx: radiotherapy; postop: postoperative; ESKD: end-stage kidney disease; NB: nephroblastomatosis; OS: overall survival; NA: not available; CPDN: cystic partially differentiated nephroblastoma; ET: epithelial type; DA: diffuse anaplasia; *: preoperative radiotherapy; ST: stromal type; y: years; (m): mean; ?: unknown number of years.

## 4. Discussion

We provide a retrospective analysis of the first five years’ experience of stage V renal tumor management in our national centralized care setting. We describe the characteristics, treatment and outcomes of this rare patient group, treated according to recent and ongoing SIOP-RTSG protocols. Our results show that although bilateral renal tumors present a therapeutic challenge, we are able to treat these patients according to SIOP-RTSG guidelines in our national center with outcomes as reported in literature [3]. With the results described here, we aimed to establish a baseline of our results from the first five years of a national centralized center, in order to evaluate our management and to accomplish future improvement of care.

Our patients with stage V Wilms tumors had a median age of 38 months, which is comparable to the unilateral Wilms tumor patients in our cohort (median 37 months) (results not shown), and to that of the stage V Wilms tumor patients in the reports listed in our literature review (Table 2). Patients with nephroblastomatosis only however, were much younger (median age 24 months), comparable to the medians of 16–31 months, reported in the largest series of nephroblastomatosis lesions in literature [60,61].

We identified hypertension at presentation in 16/24 patients (66.7%) in our series. So far, hypertension at presentation has only been reported in small series, and with a large variation between bilateral Wilms tumor patients (17–70%) (Table 2). From series with >100 patients, this information unfortunately is not available [7,24,25,26]. In Wilms tumor patients, high blood pressure has been suggested to be the result of activation of the renin-angiotensin-aldosterone system [62,63], suggesting there might be a higher risk of hypertension in patients with bilateral Wilms tumors. Therefore, monitoring and registration of blood pressure values is important in prospective clinical settings, as is treatment of hypertension to prevent complications.

Preoperative chemotherapy is recommended for bilateral Wilms tumor patients in both SIOP and COG strategies to decrease tumor size, in order to pursue maximal efforts to preserve a maximum volume of healthy kidney tissue by adequate surgery [3,64]. In our population, 26/34 Wilms tumors responded to chemotherapy, which justifies this approach. In the SIOP-93 protocol, it was observed that after preoperative chemotherapy led to the possibility of safely performing NSS in 67% of patients [28]. In our series, tumors without response turned out to be stromal (1/9 stromal tumors) or nephroblastomatosis. Progression was observed in 5/9 stromal and 1/4 diffuse anaplastic tumors (Figure 2), similar to the non-responding stage V Wilms tumors reported in the few papers in literature that reported these specific data [28,30].

When feasible, bilateral NSS is the recommended surgical strategy for stage V renal tumor patients. It has been described that renal function is better after bilateral NSS than after other types of surgery [65]. Although there may be a higher risk of positive microscopic resection margins (upstaging the tumor to local stage III), several studies have shown that positive resection margins do not necessarily lead to locoregional recurrences, when adequate postoperative therapy is applied [29,66,67]. In our series, we found microscopic positive resection margins in 2/15 (13.3%) kidneys that underwent NSS, and in 25% of our TN cases in this cohort. All patients with microscopic positive resection margins received radiotherapy. In previous reports, the percentages of positive margins after NSS in stage V patients, varied between 3% and 45% [30,33,39,44,51,68].

Postoperative radiotherapy withholding for stage V patients with local stage III in order to prevent damage to the remaining kidney tissue has been described in two reports [24,27]. These suggest that non-irradiated stage III patients have a less favorable outcome (due to relapse) than those who did receive irradiation. The first paper by Furtwängler et al. described a significantly higher progression-free survival (PFS) in irradiated local stage III patients (PFS 90%) than in non-irradiated patients (PFS 50%) and overall survival was higher in irradiated local stage III patients (90% versus 75%) [24]. The second report, by Indolfi et al., showed a high number of relapses (5/12 patients) in stage V patients with local stage III who did not receive radiotherapy [27]. Kieran et al. show that there is no difference in renal outcome between the irradiated versus non-irradiated patients [29]. In cohort, the two patients with ESKD and the patient with stage 2 CKD all received radiotherapy. However, all of these three patients also had high-risk histology and were therefore treated with intensive chemotherapy treatment and these numbers are too small to suggest any correlation. Hence, overall this suggests that radiotherapy application in stage III patients is important, however, caution is warranted and IMRT is preferred to limit the dose and field on the remnant ipsilateral kidney [23].

To diminish the occurrence of positive margins after NSS while preserving as much kidney tissue as possible, 3-D modeling, augmented reality techniques (based on preoperative imaging) and other image-based surgery options are being developed and implemented in our center [69]. Reduction of kidney mass is an important predictor for early or later development of kidney disease [70]. Since kidney disease clearly has a negative effect on normal development, as well as quality of life, preservation of kidney tissue is extremely important [27,29,67]. Unfortunately, bilateral NSS was not feasible in all patients in our series, due to size or location of the tumors. This applied to 6/23 WT patients who underwent unilateral radical nephrectomy, combined with a contralateral partial nephrectomy. This did not lead, so far, to compromised eGFR in any of these patients, although follow-up is still rather short. Fortunately, none of the 22 surviving patients had to undergo bilateral complete nephrectomies and none of these 22 surviving patients needed any kind of renal replacement therapy. Only one of these surviving patients had CKD stage 2 based on suboptimal eGFR. Nevertheless, two patients that died developed ESKD due to treatment-related toxicity, both during high-risk chemotherapy treatment, needing dialysis which started a few months after surgery. These patients experienced additional serious co-morbidity (i.e., invasive infections) which resulted in death in both patients. This illustrates the challenge of applying intensive treatment in this particular subgroup of bilateral patients, and the need for development of image-guided surgery as well as targeted therapy.

Kidney failure, early after bilateral Wilms tumor treatment varies from 0% to 54% in available reports [25,28,30,32,33,34,35,36,38,39,40,41,43,44,46,49,50,51,52,54,55,59]. Unfortunately, from the larger series, only Hamilton et al. (12%) and Montgomery et al. (5%) presented data on ESKD [25,26]. Long-term follow-up studies in unilateral Wilms tumor have shown that kidney function may still decline decades after treatment for kidney tumors [10,11,12,13,71]. Therefore, follow-up time in some reports as well as our cohort may be too short to draw strong conclusions about renal outcome. Available studies suggest that, apart from oncological surgery, nephrotoxic chemotherapy and germline predisposition contribute to this ESKD [14,72]. Awareness, prevention and monitoring of impaired kidney function already during the early treatment phase is important, since AKI is described to be associated with the development of long-term kidney failure in pediatric cancer patients [9]. In order to prevent ESKD or impaired kidney function, recommendations as defined in the ExPO-r-Net initiative, include consultation by, and/or referral to a dedicated experienced tertiary center, especially for surgery [3].

Nephroblastomatosis was present in 37/46 kidneys (78%), concomitant with Wilms tumor in 19 kidneys, and as a sole abnormality in 18 kidneys. Previous studies on bilateral Wilms tumors reported varying frequencies of nephroblastomatosis/nephrogenic rests from 19% to ‘most of their patients’. It remains challenging to distinguish nephroblastomatosis from Wilms tumor on radiology and even in pathology specimens, especially in bilateral cases [5]. Hence, nephroblastomatosis and nephrogenic rests may easily be over- or underestimated. Additionally, definitions of nephroblastomatosis and nephrogenic rests differ when comparing radiology and pathology settings. This makes it difficult to compare past and ongoing studies. Currently, a collaborative effort (HARMONICA-initiative) between COG and SIOP-RTSG takes the effort forward to harmonize endpoint definitions in histology as well as in radiology. In addition, future molecular profiling may identify specific molecular characteristics that discriminate nephrogenic rests from Wilms tumors. This may also identify nephrogenic rests that may develop into Wilms tumor over time, as was observed in three of our patients, despite a full year of chemotherapy. In general, the current advice in the SIOP-RTSG 2016 UMBRELLA protocol is to use MRI-DWI as standard of care diagnostic modality. MRI-DWI is a more sensitive tool than abdominal ultrasound in the identification of most nephroblastomatosis/nephrogenic rests at an early stage [3,73]. Ongoing efforts to pursue discrimination between nephrogenic rests and Wilms tumors based on MRI-DWI and/or liquid biopsies with specific molecular characteristics are important developments to prevent unnecessary surgery in patients with nephrogenic rests only.

Bilateral kidney tumors mostly represent Wilms tumors. We describe one (previously reported) hereditary leiomyomatosis and renal cell carcinoma (HLRCC) patient with bilateral RCC, presenting at 15 years of age. So far, we identified only eight bilateral cases of RCC in literature [74,75,76,77,78,79,80]. During our literature review, we also encountered a few other reports on pediatric kidney tumors that presented bilaterally, including malignant rhabdoid tumor of the kidney (*n* = 3) [81], clear cell sarcoma of the kidney (*n* = 2) [82], cystic nephroma (*n* = 9) [83,84,85,86,87,88,89,90,91] and CPDN (*n* = 3) [83,92,93].

Overall survival rates of large bilateral Wilms tumor/nephroblastomatosis cohorts, have been improving over the past decades [8]. In our cohort 21/23 Wilms tumor/nephroblastomatosis patients survived, and none died due to disease in these first five years. Hence, outcome seems promising, even though follow-up time (median 34 months) is rather short to draw firm conclusions from this particular series. Already during this follow-up period, three patients developed a new Wilms tumor in a previously treated nephroblastomatosis lesion, all with a confirmed Wilms tumor predisposition. We cannot exclude the possibility that a small Wilms tumor component was already present in the first place as these lesions had not been biopsied. Wilms tumor relapses that are described to occur mostly within two years after diagnosis for unilateral tumors, were not observed [16,94].

Within bilateral renal tumor patients, awareness of an underlying predisposition syndrome is important [95]. Interestingly, our genetic analyses revealed indications for tumor predisposition in 80% of the patients, more frequently than reported in a recent large meta-analysis [8]. This is conceivably explained by the fact that all patients were offered referral to a clinical geneticist as standard of care. Moreover, renal cancer genetic predisposition panel and/or whole-exome sequencing was offered in a research setting. This supports the hypothesis that patients with bilateral Wilms tumor more often suffer from genetic predisposition than unilateral Wilms tumor (~5% of patients) [5], even at a higher frequency than previously assumed. Our results underscore the importance of referral for genetic counseling in bilateral Wilms tumor patients since it affects surveillance and familial counseling and may support addition of vincristine/actinomycin-D up to one year.

## 5. Conclusions

Bilateral renal tumors represent a challenging group and induce a dilemma between attainment of oncologic complete remission and preservation of renal function. Our results describe management and the consequent favorable outcomes of patients with this rare disease in a standardized, national approach with multidisciplinary expertise. We illustrate that NSS is feasible after preoperative chemotherapy, with a low percentage of positive resection margins, leading to promising results regarding renal outcome. There still is a challenge for bilateral Wilms tumors that require intensive treatment (such as stage IV or high-risk histology), because of the risk of serious comorbidity and mortality. Detailed registries are necessary to guide future strategies for these particular subgroups. Our review of the literature shows that there is still a need to harmonize radiological and histological definitions, response criteria and recommendations for oncological, toxicity and genetic surveillance in order to compare available data for the future.

## Figures and Tables

**Table 1:** Summary of clinical information of 24 bilateral renal tumor patients.

	Number of Patients	Number of Kidneys	Number of Tumors
Total	24	48	34 Wilms tumors in 28 kidneys 39 nephrogenic rests in 37 kidneys 2 renal cell carcinoma ^1^
Sex (F/M)	13/11	-	-
Localized disease	20/24	-	-
Metastasized disease	4/24	-	-
Preoperative CT	23/24	-	-
Response to CT	-	-	26/34 Wilms tumors 18/39 nephrogenic rests
Stable disease after preoperative CT	-	-	1/34 Wilms tumors 7/39 nephrogenic rests
Progressive disease after preoperative CT	-	-	6/34 Wilms tumors 0/39 nephrogenic rests
Response to CT not available	-	-	1/34 Wilms tumors 14/39 nephrogenic rests
Surgical modality			
bNSS	4/24	-	-
uTN only	11/24	-	-
NSS + uTN	6/24	-	-
uNSS	1/24	-	-
No surgery	2/24	-	-
Histology			
Low risk	0/24	2/48	2/34
Intermediate risk	15/24	20/48	25/34 (stromal: *n* = 9)
High risk	5/24	6/48	7/34 (BT: *n* = 2, DA: *n* = 5)
Nephrogenic rest	21/24	37/39	PLNR *n* = 21 lesions ILNR *n* = 5 lesions ILNR + PLNR *n* = 4 lesions NOS *n* = 9 lesions
Nephrogenic rest only	3/24	18/48	-
Renal cell carcinoma	1/24	2/48	-
No histology available	2/24 (nephrogenic rest only based on imaging)	-	-
Postop CT	23/24	46/48	-
Postop CT+RT	7/24	-	-
Outcome			
Relapse	0 */24	-	-
Renal failure	2/24	-	-
Alive	22/24	-	-
Death	2/24	-	-

F: female; M: male; ^1^: the RCC patient had a renal cell carcinoma with a contralateral conglomerate of cysts; CT: chemotherapy; bNSS: bilateral nephron-sparing surgery, uTN: unilateral tumornephrectomy; NSS: nephron-sparing surgery; uNSS: unilateral nephron-sparing surgery; BT: blastemal type; DA: diffuse anaplasia; PLNR: perilobar nephrogenic rest; ILNR: intralobar nephrogenic rest; NOS: not otherwise specified; RCC: renal cell carcinoma; RT: radiotherapy; *: three patients developed a new Wilms tumor in a previously treated nephrogenic rest.

## Data Availability

Data is contained within the article. The data presented in this study are available in Tables S1 and S2 and in the Supplementary Materials.

## Supplementary Materials

The following are available online at https://www.mdpi.com/article/10.3390/jcm10235558/s1, Table S1: Search terms, Table S2: Detailed information of 24 patients with bilateral renal tumors., Figure S1: PRISMA flow diagram of article selection, Table S3: Presenting symptoms, Table S4: Stage V renal tumor patients with metastatic disease.
