# Supplementary material for: Bilateral Renal Tumors in Children: The First 5 Years’ Experience of National Centralization in The Netherlands and a Narrative Review of the Literature
Source: J Clin Med. 2021 Nov 26;10(23):5558. doi: 10.3390/jcm10235558 (PMC8658527; doi:10.3390/jcm10235558)
Supplement: Supplementary file 1 [file jcm-10-05558-s001.zip › jcm-1437133-supplementary.pdf]

Table S1. Search terms

**Embase**	(bilateral:ab,ti OR (stage AND v:ab,ti) OR (stage AND 5:ab,ti)) AND (wilm*:ab,ti OR nephroblastom*:ab,ti)<br>(bilateral;ab,ti OR (stage AND v:ab,ti) OR (stage AND 5:ab,ti)) AND (CCSK:ab,ti OR clear cell sarcoma of the kidney:ab,ti)<br>(bilateral:ab,ti OR (stage AND v:ab,ti) OR (stage AND 5:ab,ti)) AND (MRTK:ab,ti OR malignant rhabdoid tumor of the kidney:ab,ti)<br>(bilateral:ab,ti OR (stage AND v:ab,ti) OR (stage AND 5:ab,ti)) AND (RCC:ab,ti OR renal cell carcinoma:ab,ti)<br>(bilateral:ab,ti OR (stage AND v:ab,ti) OR (stage AND 5:ab,ti)) AND (CN:ab,ti OR cystic nephroma:ab,ti)<br>(bilateral:ab,ti OR (stage AND v:ab,ti) OR (stage AND 5:ab,ti)) AND (CPDN:ab,ti OR cystic partially differentiated nephroblastoma:ab,ti)<br>(bilateral:ab,ti OR (stage AND v:ab,ti) OR (stage AND 5:ab,ti)) AND (CMN:ab,ti OR MN:ab,ti) OR (mesoblastic nephrom*:ab,ti))
**PubMed**	("bilateral"[Title/Abstract] OR "stage v"[Title/Abstract] OR "stage 5"[Title/Abstract]) AND ("wilm*"[Title/Abstract] OR "nephroblastom*"[Title/Abstract])<br>("bilateral"[Title/Abstract] OR "stage v"[Title/Abstract] OR "stage 5"[Title/Abstract]) AND ("CCSK"[Title/Abstract] OR " clear cell sarcoma of the kidney "[Title/Abstract])<br>("bilateral"[Title/Abstract] OR "stage v"[Title/Abstract] OR "stage 5"[Title/Abstract]) AND ("MRTK"[Title/Abstract] OR "malignant rhabdoid tumor of the kidney"[Title/Abstract])<br>("bilateral"[Title/Abstract] OR "stage v"[Title/Abstract] OR "stage 5"[Title/Abstract]) AND ("RCC"[Title/Abstract] OR "renal cell"[Title/Abstract]) AND ("child*"[Title/Abstract] OR "pediatr*"[Title/Abstract])<br>("bilateral"[Title/Abstract] OR "stage v"[Title/Abstract] OR "stage 5"[Title/Abstract]) AND ("CN"[Title/Abstract] OR "cystic nephrom*"[Title/Abstract]) AND ("child*"[Title/Abstract] OR "pediatr*"[Title/Abstract])<br>("bilateral"[Title/Abstract] OR "stage v"[Title/Abstract] OR "stage 5"[Title/Abstract]) AND ("CPDN"[Title/Abstract] OR "cystic partially differentiated nephroblastom*"[Title/Abstract])<br>("bilateral"[Title/Abstract] OR "stage v"[Title/Abstract] OR "stage 5"[Title/Abstract]) AND ("CMN"[Title/Abstract] OR "MN"[Title/Abstract] OR "mesoblastic nephrom*")

Table S2. Detailed information of 24 patients with bilateral renal tumors.

Pt No.	Sex	Age (mths)	Metastasized disease	HTN at Dx	Preop CT	Tumor volume response (%)		Surgery type		Local stage		Tumor histology		Postop therapy		Outcome		eGFR last FU (ml/min/ 1.73m²)	FU (mths)
						L	R	L	R	L	R	L	R	CT	RTx	Relapse	Death		
**1**	F	44	No	No	VA	-91	-93	NSS	NSS	I	III	RT-WT	RT-WT	Yes	Yes	No	No	>90	74
						NA	NA					NB (PLNR)	NB (PLNR)						
**2**	M	46	No	Yes	VA-D	-93	-73	NSS	TN	I	I	RT-WT	RT-WT	Yes	No	No	No	>90	66
**3**	M	52	No	Yes	VA	-87	-66	NSS	TN	III	I	BT-WT	ST-WT	Yes	Yes	No	Yes	<15	20 (died)
						-100	NA					RT-WT	NB (PLNR)						
**4**	F	11	No	Yes	VA-CE	+43	-30.5	TN	NSS (3y later)	I	III^	ST-WT	ST-WT (2nd tumor)	Yes	No	No#	No	>90	58
							-6.5						NB (ILNR)						
**5**	F	56	No	No	VA	-90	-95	B	TN	I	II	CNT-WT	DA-WT	Yes	No	No	No	>90	51
							-33						RT-WT						
						NA	-15					NB (PLNR)	TLNR*						
							NA						NB (PLNR)						
**6**	M	57	Yes (osseous Th10)	Yes	VAD	-78	-77	NSS	NSS	III	I	RT-WT	CNT-WT	Yes	Yes	No	No	>90	33
						Stable	-80					NB (PLNR)	NB (PLNR)						
**7**	F	59	Yes (pulmonary)	Yes	VAD	-79	-98	TN	NSS	III	I	DA-WT	RT-WT	Yes	Yes	No	No	89	27
						-67	NA					DA-WT	NB (PLNR)						
						NA						NB (PLNR)							
**8**	M	49	Yes (pulmonary)	Yes	VA-D	+29	-59	TN	None	III	NA	DA-WT	DA-WT	Yes	Yes	No	Yes	<15	12 (died)
**9**	F	15	No	Yes	VA-D	-77	-91	NSS	NSS	NA	I	PLNR*	BT-WT	Yes	No	No	No	>90	34
							NA						NB (PLNR)						

**10**	F	21	No	Yes	VA	-100	-91<br>-97<br>-94<br>-100	None	TN	NA	III	PLNR[1]	RT-WT<br>RT-WT<br>NB (PLNR)<br>PLNR	Yes	Yes	No	No	>90	63
**11**	F	54	No	No	VA	-78<br>NA	↓[3]	TN	B	I	NA	RT-WT<br>PLNR	NR*	Yes	No	No	No	>90	49
**12**	F	51	No	Yes	VA	-75<br>NA<br>NA	>-10[2]	TN	NSS	II	NA	RT-WT<br>RT-WT<br>NB (PLNR)	PLNR*	Yes	No	No	No	>90	48
**13**	F	38	No	Yes	VA	Stable	-71<br>NA	B	TN	NA	I	NB	RT-WT<br>NB (PLNR+ILNR)	Yes	No	No	No	>90	60
**14**	M	9	No	Yes	VA-D	-1.4	-60	TN	NSS	I	NA	ST-WT	NB (ILNR)*	Yes	No	No	No	>90	33
**15**	F	12	No	Yes	VA-CE	↓[3]	+164<br>+48.2	None	TN	II	NA	NR[1]	ST-WT<br>ST-WT	Yes	No	No	No	>90	29
**16**	M	19	No	No	VA	-90<br>↓[3]	↓[3]	NSS	None	NA	I	MT-WT<br>NB (PLNR)	NB (PLNR)[1]	Yes	No	No[#]	No	>90	28
**17**	F	24	No	No	VA	-77<br>NA	Stable	TN	None	II	NA	ST-WT<br>ILNR	NB (ILNR)[1]	Yes	No	No	No	>90	21
**18**	M	7	No	Yes	VA	Stable	+59<br>NA	None	TN	NA	I	NB[1]	ST-WT<br>ILNR	Yes	No	No[#]	No	>90	19
**19**	F	51	Yes (m. psoas + pulmonary + osseous Th12)	Yes	VAD	Stable	-91	None	TN	NA	III	NB[1]	RT-WT	Yes	Yes	No	No	>90	36
**20**	M	17	No	Yes	VA	-34.5	+145	None	TN	NA	III	NB[1]	ST-WT	Yes	Yes	No	No	>90	32

							Stable						NB (PLNR+ILNR)						
**21**	M	12	No	Yes	VA	↓[3]	↓[3]	None	B	NA		NB[1]	NB[1]	NA	No	No	No	>90	36
**22**	M	24	No	No	VA	>-10[2]	-97	NSS	NSS	NA		NB (PLNR)	NB (PLNR)	Yes	No	No	No	>90	23
**23**	M	25	No	Yes	VA	Stable	Stable	None	None	NA		NB[1] (PLNR+ILNR)	NB[1] (PLNR+ILNR)	NA	No	No	No	>90	62
**24**	F	184	No	No	No	NA	NA	None	TN	NA	I	NA	Papillary RCC type 2	No	No	No	No	>90	60

Pt. no: patient number; mths: months; Dx: diagnosis; M: male; F: female; HTN: hypertension; NSS: nephron sparing surgery; TN: tumornephrectomy; B: biopsy; RT: regressive type; ST: stromal type; BT: blastemal type; DA: diffuse anaplastic; CNT: completely necrotic type; MT: mixed type; WT: Wilms tumor; NB: nephroblastomatosis; NR: nephrogenic rest; PLNR: perilobar nephrogenic rest; ILNR: intralobar nephrogenic rest; TLNR: tumor like nephrogenic rest; preop: preoperative; postop: postoperative; CT: chemotherapy; RTx: radiotherapy; eGFR: estimated glomerular filtration rate; VA: vincristine/actinomycin-D; VAD: vincristine/actinomycin-D/doxorubicin; VA-D: vincristine/actinomycin-D/addition of doxorubicin for stable disease; VA-CE: vincristine/actinomycin-D/addition of carboplatin/etoposide for stable disease; [1]: no histological confirmation of diagnosis; Th: thoracic vertebra; ^:only nephrogenic rest in resection margins, no Wilms tumor cells; [#]: development of Wilms tumor during follow-up in a previously completely treated (suspected) nephroblastomatosis lesion; *: diagnosis of nephroblastoma cannot be excluded, but nephrogenic rest is preferred diagnosis; [2]: Volume after chemotherapy was too small for reliable volume assessment; [3]: decrease in size of the nephrogenic rest, dimensions were not specified; FU: follow-up; NA: not applicable; RCC: renal cell carcinoma

Table S3. Presenting symptoms

**Presenting symptom**	**Frequency (%)**
Abdominal mass	14 (58%)
Abdominal pain	6 (25%)
Asymptomatic	6 (25%)
Surveillance for underlying syndrome	5 (21%)
Incidental finding upon traumatic workup	1 (4%)
Fever	2 (8%)
Loss of appetite	2 (8%)
Hematuria	1 (4%)
Fatigue	1 (4%)
Constipation	1 (4%)
Headache	1 (4%)

Figure S1. PRISMA flow diagram of article selection

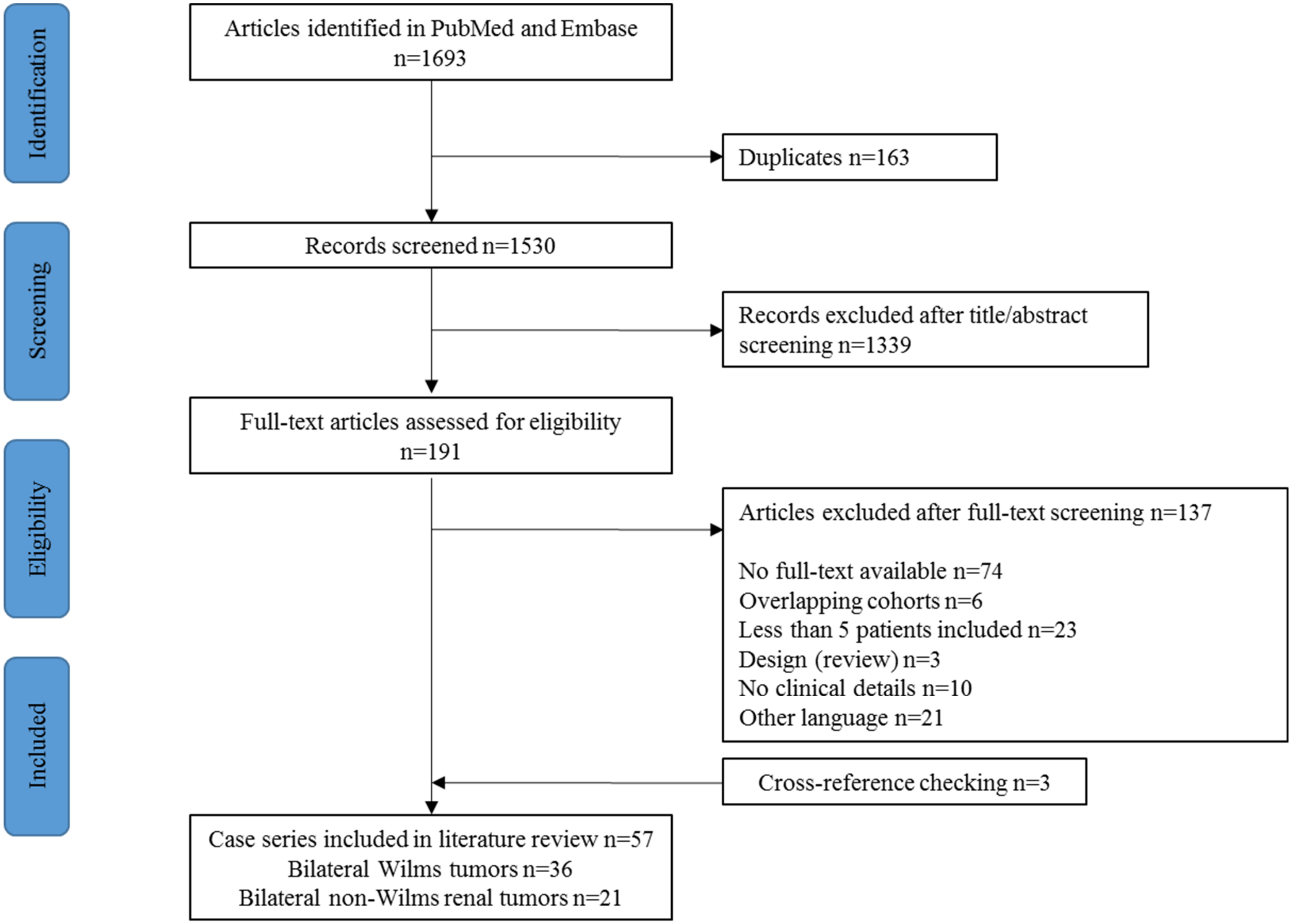

Table S4. Stage V renal tumor patients with metastatic disease

Author	No. of patients with metastasis	Histology	Outcome (Histology)
**Aydin 2019 [1]**	4	NA	NA
**Ehrlich 2017 [2]**	27	3 anaplasia n.o.s.	NA (at least 3 anaplasia died)
**Davidoff 2015 [3]**	7	At least 1 DA<br>At least 1 FH n.o.s.	2/7 died (DA and FH)
**Hubertus 2015 [4]**	4	2 blastemal, 2 NA	2/4 died (blastemal)
**Agarwala 2014 [5]**	3	NA	NA
**Furtwängler 2014 [6]**	19	NA	NA
**Oue 2014 [7]**	2	NA	NA
**Hadley 2013 [8]**	4	NA	NA
**Indolfi 2013 [9]**	11	NA (No DA)	NA
**Sulkowski [10]**	1	NA	NA
**Sudour 2012 [11]**	5	NA	NA
**Halim 2012 [12]**	5	3 UH n.o.s.<br>2 FH n.o.s.	5 (3 UH, 2 FH)
**Millar 2011 [13]**	4	NA	4/4 died
**Hamilton 2011 [14]**	16	NA	NA
**Weirich 2004 [15]**	3	NA	NA
**Kubiak 2004 [16]**	4	NA	NA
**Tomlinson 1999 [17]**	1	NA (No DA)	1/1 died (NA)
**Kumar 1998 [18]**	9	NA	NA
**Shearer 1993 [19]**	4	NA	NA
**Montgomery 1991 [20]**	35	NA	NA
**Coppes 1989 [21]**	5	NA	NA
**Cohen 1986 [22]**	4	NA	NA
**Asch 1985 [23]**	3	NA (No DA)	2/3 died (NA)
**Jones 1982 [24]**	4	NA (No DA)	4/4 died (NA)
**This cohort, 2021**	4	1 Regressive bilateral	1/4 died (DA)

		1 Blastemal + stromal<br>2 bilateral DA	
**Total**	188	162 NA, 16 specified	24/35 died

No.: number; NA: not available; DA: diffuse anaplasia; FH: favorable histology; n.o.s.: not otherwise specified; UH: unfavorable histology.

References

1. Aydin, B.; Akyuz, C.; Yalcin, B.; Ekinci, S.; Oguz, B.; Akcoren, Z.; Yildiz, F.; Varan, A.; Kurucu, N.; Buyukpamukcu, M.; et al. Bilateral Wilms tumors: Treatment results from a single center. *Turk. J. Pediatr.* **2019**, *61*, 44-51, doi:10.24953/turkjped.2019.01.008.
2. Ehrlich, P.; Chi, Y.Y.; Chintagumpala, M.M.; Hoffer, F.A.; Perlman, E.J.; Kalapurakal, J.A.; Warwick, A.; Shamberger, R.C.; Khanna, G.; Hamilton, T.E.; et al. Results of the First Prospective Multi-institutional Treatment Study in Children With Bilateral Wilms Tumor (AREN0534): A Report From the Children's Oncology Group. *Ann. Surg.* **2017**, *266*, 470-478, doi:10.1097/SLA.0000000000002356.
3. Davidoff, A.M.; Interiano, R.B.; Wynn, L.; Delos Santos, N.; Dome, J.S.; Green, D.M.; Brennan, R.C.; McCarville, M.B.; Krasin, M.J.; Kieran, K.; et al. Overall Survival and Renal Function of Patients With Synchronous Bilateral Wilms Tumor Undergoing Surgery at a Single Institution. *Ann. Surg.* **2015**, *262*, 570-576, doi:10.1097/SLA.0000000000001451.
4. Hubertus, J.; Gunther, B.; Becker, K.; Graf, N.; Furtwangler, R.; Ferrari, R.; Gruhn, B.; Stahl, R.; von Schweinitz, D.; Stehr, M. Development of hypertension is less frequent after bilateral nephron sparing surgery for bilateral Wilms tumor in a long-term survey. *J. Urol.* **2015**, *193*, 262-266, doi:10.1016/j.juro.2014.07.116.
5. Agarwala, S.; Mittal, D.; Bhatnagar, V.; Srinivas, M.; Bakhshi, S.; Bajpai, M.; Gupta, D.K.; Iyer, V.K.; Mohanti, B.K.; Thulkar, S. Management and outcomes in massive bilateral Wilms' tumors. *J. Indian Assoc. Pediatr. Surg.* **2014**, *19*, 208-212, doi:10.4103/0971-9261.142005.
6. Furtwangler, R.; Schmolze, M.; Graber, S.; Leuschner, I.; Amann, G.; Schenk, J.P.; Niggli, F.; Kager, L.; von Schweinitz, D.; Graf, N. Pretreatment for bilateral nephroblastomatosis is an independent risk factor for progressive disease in patients with stage V nephroblastoma. *Klin. Padiatr.* **2014**, *226*, 175-181, doi:10.1055/s-0034-1371840.
7. Oue, T.; Koshinaga, T.; Okita, H.; Kaneko, Y.; Hinotsu, S.; Fukuzawa, M. Bilateral Wilms tumors treated according to the Japan Wilms Tumor Study Group protocol. *Pediatr. Blood Cancer* **2014**, *61*, 1184-1189, doi:10.1002/pbc.24979.
8. Hadley, G.P.; Mars, M.; Ramdial, P.K. Bilateral Wilms' tumour in a developing country: a descriptive study. *Pediatr. Surg. Int.* **2013**, *29*, 419-423, doi:10.1007/s00383-013-3287-7.
9. Indolfi, P.; Jenkner, A.; Terenziani, M.; Crocoli, A.; Serra, A.; Collini, P.; Biasoni, D.; Gandola, L.; Bisogno, G.; Cecchetto, G.; et al. Synchronous bilateral Wilms tumor: a report from the Associazione Italiana Ematologia Oncologia Pediatrica (AIEOP). *Cancer* **2013**, *119*, 1586-1592, doi:10.1002/cncr.27897.
10. Sulkowski, J.; Kolon, T.; Mattei, P. Nephron-sparing partial nephrectomy for bilateral Wilms' tumor. *J. Pediatr. Surg.* **2012**, *47*, 1234-1238, doi:10.1016/j.jpedsurg.2012.03.032.
11. Sudour, H.; Audry, G.; Schleimacher, G.; Patte, C.; Dussart, S.; Bergeron, C. Bilateral Wilms tumors (WT) treated with the SIOP 93 protocol in France: epidemiological survey and patient outcome. *Pediatr. Blood Cancer* **2012**, *59*, 57-61, doi:10.1002/pbc.24059.
12. Halim, A.; Elashry, R.; Soliman, O.; Sarhan, O. Bilateral Wilms' tumor: Mansoura multi-centers 15 years experience. *J. Oncol. Pharm. Pract.* **2012**, *18*, 115-121, doi:10.1177/1078155210396575.
13. Millar, A.J.W.; Davidson, A.; Rode, H.; Numanoglu, A.; Hartley, P.S.; Desai, F. Nephron-sparing surgery for bilateral Wilms' tumours: a single-centre experience with 23 cases. *Afr. J. Paediatr. Surg.* **2011**, *8*, 49-56, doi:10.4103/0189-6725.78669.
14. Hamilton, T.E.; Ritchey, M.L.; Haase, G.M.; Argani, P.; Peterson, S.M.; Anderson, J.R.; Green, D.M.; Shamberger, R.C. The management of synchronous bilateral Wilms tumor: a report from the National Wilms Tumor Study Group. *Ann. Surg.* **2011**, *253*, 1004-1010, doi:10.1097/SLA.0b013e31821266a0.
15. Weirich, A.; Ludwig, R.; Graf, N.; Abel, U.; Leuschner, I.; Vujanic, G.M.; Mehls, O.; Boos, J.; Beck, J.; Royer-Pokora, B.; et al. Survival in nephroblastoma treated according to the trial and study SIOP-9/GPOH with respect to relapse and morbidity. *Ann. Oncol.* **2004**, *15*, 808-820, doi:10.1093/annonc/mdh171.
16. Kubiak, R.; Gundeti, M.; Duffy, P.G.; Ransley, P.G.; Wilcox, D.T. Renal function and outcome following salvage surgery for bilateral Wilms' tumor. *J. Pediatr. Surg.* **2004**, *39*, 1667-1672, doi:10.1016/j.jpedsurg.2004.07.009.
17. Tomlinson, G.S.; Cole, C.H.; Smith, N.M. Bilateral Wilms' tumor: a clinicopathologic review. *Pathology* **1999**, *31*, 12-16, doi:10.1080/003130299105458.
18. Kumar, R.; Fitzgerald, R.; Breatnach, F. Conservative surgical management of bilateral Wilms tumor: results of the United Kingdom Children's Cancer Study Group. *J. Urol.* **1998**, *160*, 1450-1453, doi:10.1016/s0022-5347(01)62588-6.

19. Shearer, P.; Parham, D.M.; Fontanesi, J.; Kumar, M.; Lobe, T.E.; Fairclough, D.; Douglass, E.C.; Wilimas, J. Bilateral Wilms tumor. Review of outcome, associated abnormalities, and late effects in 36 pediatric patients treated at a single institution. *Cancer* **1993**, *72*, 1422-1426, doi:10.1002/1097-0142(19930815)72:4<1422::aid-cncr2820720443>3.0.co;2-e.
20. Montgomery, B.T.; Kelalis, P.P.; Blute, M.L.; Bergstralh, E.J.; Beckwith, J.B.; Norkool, P.; Green, D.M.; D'Angio, G.J. Extended Followup of Bilateral Wilms Tumor: Results of the National Wilms Tumor Study. *J. Urol.* **1991**, *146*, 514-518, doi:10.1016/s0022-5347(17)37840-0.
21. Coppes, M.J.; de Kraker, J.; van Dijken, P.J.; Perry, H.J.; Delemarre, J.F.; Tournade, M.F.; Lemerle, J.; Voûte, P.A. Bilateral Wilms' tumor: long-term survival and some epidemiological features. *J. Clin. Oncol.* **1989**, *7*, 310-315, doi:10.1200/JCO.1989.7.3.310.
22. Cohen, R.C.; Kelly, J.H.; Chan, H.S.L.; Mancer, K.; Weitzman, S.; Daneman, A.; Filler, R.M. Treatment and prognosis of 32 patients with bilateral Wilms' tumor. *Pediatr. Surg. Int.* **1986**, *1*, 110-116, doi:10.1007/BF00166871.
23. Asch, M.J.; Siegel, S.; White, L.; Fonkalsrud, E.; Hays, D.; Isaacs, H. Prognostic Factors and Outcome in Bilateral Wilms ' Tumor. *Cancer* **1985**, *56*, 2524-2529, doi:10.1002/1097-0142(19851115)56:10<2524::aid-cncr2820561034>3.0.co;2-x.
24. Jones, B.; Hrabrovski, E.; Kiviat, N.; Breslow, N.E. Metachronous bilateral Wilms' tumor, National Wilms' Tumor Study. *Am. J. Clin. Oncol.* **1982**, *5*, 545-550.
